# Application of U-FAST Technology in Sintering of Submicron WC-Co Carbides

**DOI:** 10.3390/ma16062450

**Published:** 2023-03-19

**Authors:** Joanna Wachowicz, Robert Kruzel, Zbigniew Bałaga, Agnieszka Ostrowska, Tomasz Dembiczak

**Affiliations:** 1 Institute of Wood Sciences and Furniture, Department of Mechanical Processing of Wood, Warsaw University of Life Sciences, Nowoursynowska Street, 166, 02-787 Warsaw, Poland; 2 Faculty of Civil Engineering, Czestochowa University of Technology, Akademicka Street 3, 42-201 Czestochowa, Poland; 3 Faculty of Production Engineering and Materials Technology, Czestochowa University of Technology, Armii Krajowej Street, 19, 42-201 Czestochowa, Poland; 4 Department of Nanobiotechnology, Institute of Biology, Warsaw University of Life Sciences, Ciszewskiego Street 8, 02-786 Warsaw, Poland; 5 Faculty of Science and Technology, Jan Dlugosz University in Czestochowa, Armii Krajowej Street 13/15, 42-200 Czestochowa, Poland

**Keywords:** powder metallurgy, sintering, WC-Co carbides

## Abstract

This article presents the microstructure, hardness, fracture toughness coefficient *K_IC_* and phase composition of submicron WC-4Co carbides. The carbides were sintered using the innovative U-FAST (Upgraded Field Assisted Sintering Technology) method, from mixtures of WC-Co powders with an average WC grain size of 0.4 µm and 0.8 µm. The obtained sinters were characterized by a relative density above 99% of the theoretical density. The hardness of the obtained composites was above 2000 HV30, while the *K_IC_* coefficient was about 8 MPa m^1/2^.

## 1. Introduction

Sintered WC-Co carbides are materials which are characterized by high wear resistance and heat resistance, and have high hardness with relatively high toughness. Due to their properties, they are widely used in tooling applications related to machining, cutting and drilling. WC-Co composites consist of a hard, hexagonal WC phase and soft Co binder [1,2,3,4]. Cobalt is the most commonly used binder as it has high bending strength, very good thermal conductivity and satisfactory wettability (wetting angle of approximately 0°). Moreover, cobalt shows ferromagnetic properties. It has an HCP (hexagonal close-packed) structure and undergoes an allotropic transformation to FCC (face-centered cubic) at 450 °C. The properties of cobalt make it a very good material to use as a matrix. In addition, the melting point of cobalt (T_melt._Co = 1494 °C) is much lower than that of the tungsten carbide (T_melt._WC = 2785 °C), which makes it possible to obtain sintered materials at low temperatures, as well as limiting the growth of WC grains [5,6,7]. Apart from the WC grain size, the cobalt content plays an important role, influencing the hardness and durability of carbide blades, at the same time.

Sintered carbides are obtained by powder metallurgy [8,9,10,11]. Powder metallurgy technology is characterized by a number of advantages. One of them is the high utilization of raw materials (up to 95%). Thanks to the elimination of machining, it is regarded as a material-saving technology. Another advantage of powder metallurgy is the wide range of adjustment of the chemical composition, sinter properties and structure. Unfortunately, when analyzing this technology, some disadvantages can be encountered, including the difficulty in obtaining complex-shaped material due to uneven pressure distribution during pressing [12,13,14].

For conventional sintering of WC-Co carbides, vacuum, induction or resistance furnaces are used. The suitably mixed powder mixture is subjected to densification, pre-sintering and final sintering in the liquid phase at a temperature of about 1300–1500 °C. The sintering process involves the dissolution of WC grains in the cobalt bonding phase, followed by migration and re-precipitation on the surface of the original WC phase. At the eutectic temperature, about 12% of the tungsten carbide is dissolved in the bonding phase. As the temperature decreases, the solubility of WC decreases to about 1% in the range of # 800–600 °C [15,16].

In recent years, there has been growing interest in modern sintering methods, referred to as the FAST (Field Assisted Sintering Technology) methods, due to increasing customer expectations of reduced sintering time and energy efficiency. An example of such technology is SPS (Spark Plasma Sintering), which uses electrical pulses to heat the powder mixture [17,18,19,20]. The powder, which undergoes the sintering, is placed in a graphite set-up consisting of a die and punches. Use of this material is not accidental, as graphite has very good thermal and electrical conductivity. The direct current pulses used in this method reach currents of up to 10 kA [21,22,23,24]. The duration of such a pulse ranges from a few to several hundred milliseconds. The electric current flowing between the powder particles causes an electrical discharge, a rise in temperature in the micro-areas, as well as the over-melting of the material resulting in the formation of necks.

The aim of the work was to fabricate WC-Co composites by U-FAST technology, and to determine the influence of used method on the microstructure, phase composition and fracture toughness of the obtained sinters.

## 2. Experimental Procedure

In this study, the submicron WC-4Co cemented carbides were fabricated using a new U-FAST method. The influence of the fabrication technology on the microstructure, phase composition as well as hardness and fracture toughness of WC-4Co sintered carbides were investigated. The results obtained were compared with similar sintered alloys obtained by SPS technology.

The WC-Co powders were sintered in a U-FAST device (GeniCore, Warsaw, Poland). The advantage of the FAST technology is that the powder materials consolidation process is carried out at a much lower temperature than traditional methods available in the industry. This reduces grain growth during the sintering process, and at the same time, ensures better properties of the obtained materials. In addition, the key aspect of FAST is high energy efficiency associated mainly with the direct generation of heat inside the material, which positively affects the homogeneity of the created materials and significantly reduces the sintering time [25,26]. The device parameters are shown in Table 1. In order to exploit all the advantages of FAST/SPS technology effectively, the new U-FAST (upgraded Field Assisted Sintering Technology) device uses short-duration electrical pulses, with a voltage several tens of times higher than that of other typical SPS devices, which opens up the prospect of sintering for new materials.

The following parameters of the sintering process were used. The temperatures and holding times were selected experimentally. The sintering temperature was 1220 °C with holding times of 10 min. This process occurred under a load of 100 MPa. Cooling the samples in a vacuum of 10–6 mbar was the last stage. The sinters were then subjected to a mechanical treatment consisting of a two-side surface polishing to a sample thickness of 2 mm. The diameter of the WC-Co sinters was 20 mm.

The density of the sintered alloys was measured using the Archimedes method. The microstructure of specimens was studied using an SEM–FEI QUANTA 200, and X-ray diffraction (XRD) analysis was carried out for phase identification. 

Grain size analysis was performed on the basis of images of the surface of the samples, using the Image Pro Plus software.

The roughness of the samples was measured with the Mitutoyo Surf Test SJ-201 device. The measured distance was 0.8 mm. On each blade, three measurements were taken, and the following were determined: the arithmetic mean deviation of the profile from the mean line—Ra, the height of roughness following ten points of the profile—Rz, and the quadratic mean deviation of the profile from the mean line along the measured section—Rq.

Hardness was measured using a Vickers hardness tester under a constant load of 30 kg. Hardness measurements were made along the diameter of the sintered samples. For the fracture toughness (*K_IC_*), the radial crack length around the Vickers indentation was measured and the Shetty formula was applied [28], according to:

(1) $$K_{IC}=A\sqrt{H}\left(\frac{P}{\sum^{}L}\right)$$

where *H* is the hardness (N/mm^2^), *P* is the applied load (N), ∑*L* is the sum of crack lengths (mm), *A* is a constant with a value of 0.0028 and *K_IC_* is given as MPa m^1/2^. For HV30 values expressed in (kgf/mm^2^), Palmqvist fracture toughness can be calculated as:

(2) $$K_{IC}=0.15\left(\frac{HV_{30}}{\sum^{}L}\right)$$

## 3. Characterization of Starting Material

**Figure 1:**
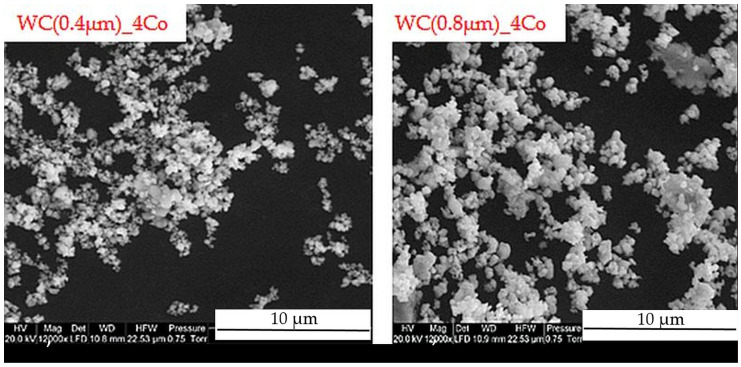
SEM micrograph of starting WC-4Co powder.

Mixtures of WC and Co powders were used for the consolidation process. The cobalt content was 4% by weight, while the average WC grain size, as specified by the manufacturer, was 0.4 µm and 0.8 µm. Figure 1 shows SEM images of the WC-4Co powder mixtures used in the study. The powders used are characterized by their irregular shape and form agglomerates of different sizes. It is important that the Co is homogeneously dispersed in the mixture. This will not only facilitate the compacting of the powder particles, but can also improve the performance during sintering by directing a continuous bridge of Co between the WC. Correct mixture preparation is particularly important for the sintering process, also due to the absence of WC grain growth inhibitors.

An analysis of the chemical composition of the powders to be sintered, enclosed by the supplier, is shown in Table 2 and Table 3. 

**Figure 2:**
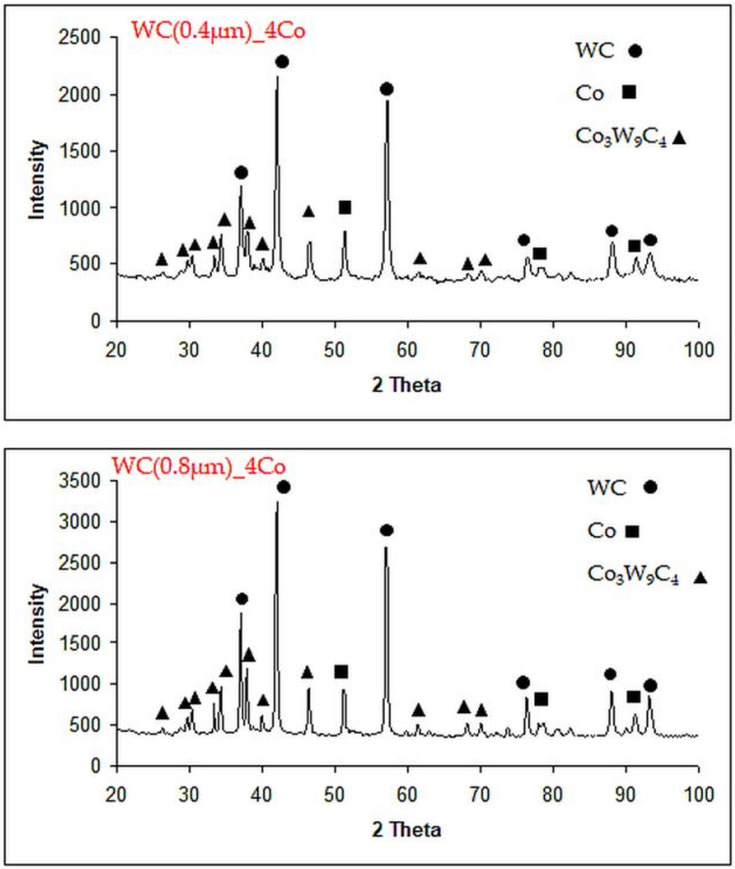
XRD patterns with a selected 2θ range of 20°–100° for WC-4Co powders (Co: 96-901-2885; WC: 96-210-2245; Co_3_W_9_C_4_: 96-152-8858).

Diffraction analysis of the powder mixture showed the presence of three phases: WC, Co and Co_3_W_9_C_4_ (Figure 2).

## 4. Results and Discussion

**Figure 3:**
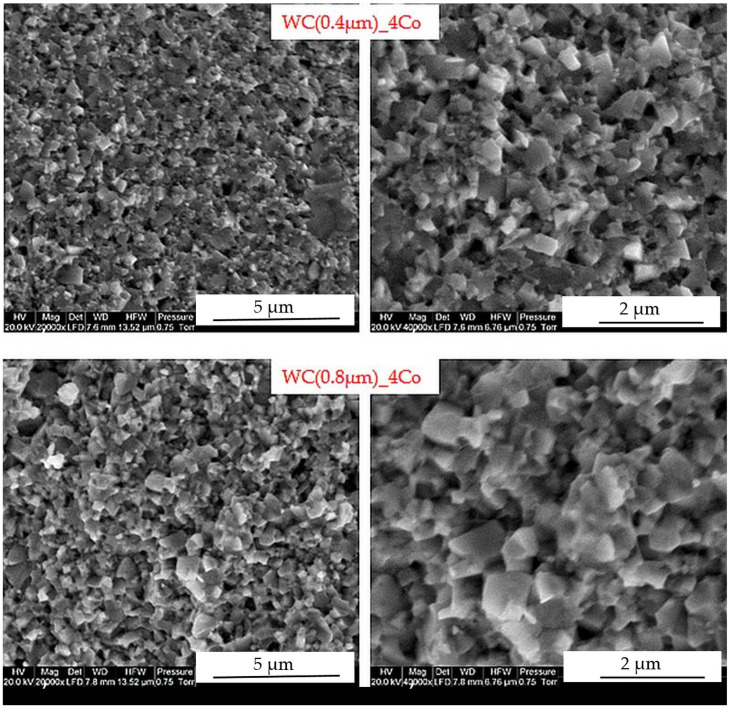
SEM (SE) micrographs of microstructure with sintered WC-Co composites.

The microstructure of WC-4Co cemented carbide fractures with different WC sizes was observed using the secondary electron (SE) mode in the SEM, as shown in Figure 3. WC grains are irregular polygons. No cobalt clusters were observed. In general, no clearly abnormal growth of WC particles was found in the carbides at the final sintering temperature, although individual micrometre-sized grains were observed.

**Figure 4:**
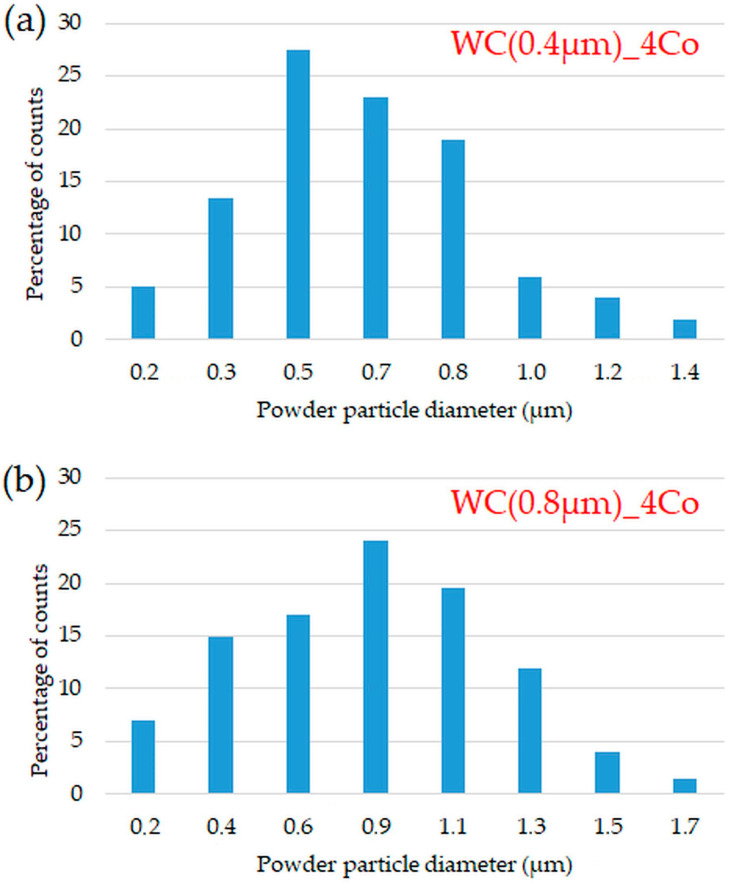
Particle size distribution for sintered WC-4Co for grain sizes of the starting WC powder: (**a**) 0.4 µm; (**b**) 0.8 µm.

Figure 4 presents histograms showing the grain size distribution of the obtained WC-4Co composites, for different grain sizes of the starting WC powder: 0.4 µm (Figure 4a); 0.8 µm (Figure 4b). When sintering samples from WC powder with a grain size gradation of 0.4 µm, WC grains with an average diameter in the range 0.5–0.8 µm accounted for the largest proportion of grains—about 70%. It should be noted, however, that the analysis showed, approximately, a 10% proportion of WC grains with a diameter greater than 1 µm. 

In contrast, for samples sintered from 0.8 µm powder, grains of 0.5–0.8 µm accounted for 40%. In both cases, the average grain size was slightly larger compared to the original WC powder.

In similar studies, in works [29,30,31] it was observed that although ultrafine-grained sintered carbides (average WC size: 0.1–0.6 μm) have higher hardness and wear resistance, their fracture resistance is worse than that of coarse-grained carbides, which may affect their use in the tooling industry.

Therefore, research is being conducted, among other things, into the introduction of a micrograin WC additive in ultrafine carbides to improve the fracture toughness of ultrafine carbides and to achieve a combination of adequate hardness and fracture toughness [32].

In the experiment carried out, it is clear that the average grain sizes are within the range of the submicron values, which is a positive aspect. It is well known that WC grain growth in the liquid phase sintering occurs by Ostwald ripening, with the dissolution of smaller WC grains and precipitation on larger grains in liquid Co. This growth behavior is predominantly interface controlled [33]. In the case of obtaining WC-Co composites by the conventional method, a clear grain growth occurs. This phenomenon is favored by the high temperature of the process and a long time. In addition, the large surface energy of the fine powder mixture accelerates the grain growth during consolidation. In contrast, in the U-FAST method used, the sintering process takes place at a much lower temperature and in a shorter time, which significantly inhibits grain growth.

Furthermore, it should be emphasized that with the increase in Co content, the dissolution-precipitation of the grain was gradually strengthened in addition to the combined growth of the grain. The process is as follows: firstly, the sharp corner of a WC grain and a smaller grain begin to dissolve; then, WC precipitates up to form rounded corners of large grains. The disappearance of the crystal’s sharp corners could eliminate the concentration of stress at the sharp corners and improve the mechanical properties [34].

The roughness parameters of all tested sample variants were similar: Ra = 0.09 μm, Rz = 0.76 μm and Rq = 0.12 μm.

**Figure 5:**
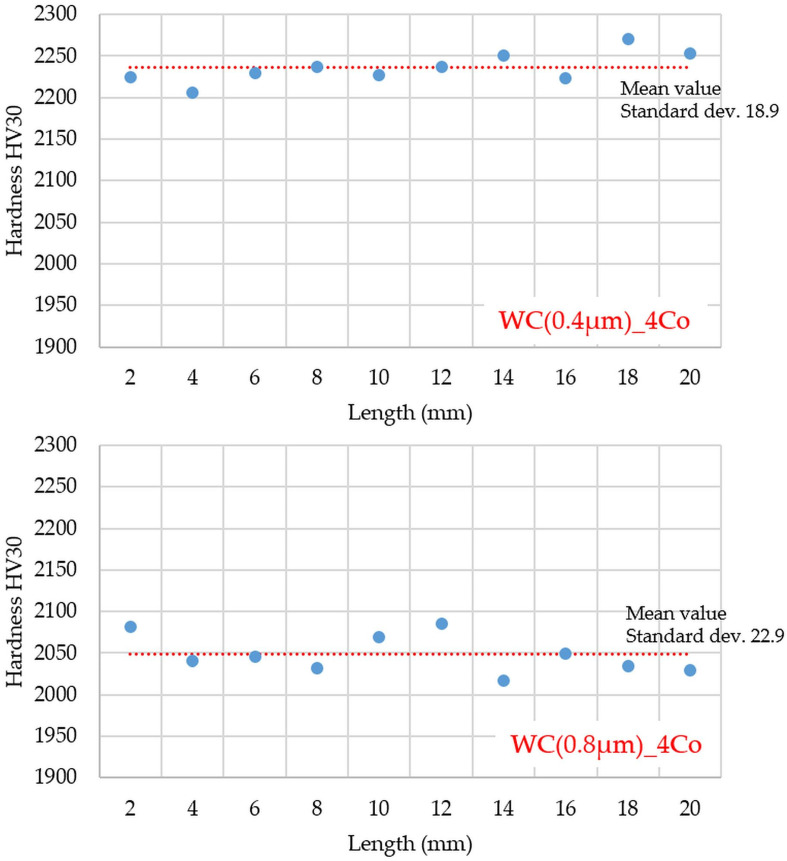
HV30 hardness profile (The blue circles mean the HV30 hardness and the red line means the average value of hardness).

All samples tested had a relative density above 99% of the theoretical density. The Vickers hardness (HV30) of the WC-4Co cemented carbides was measured at room temperature. An example of the hardness profile (the length profile was 20 mm) for the different sample variants is shown in Figure 5. As expected, the hardness of the samples increased with a reduction in WC grain size. The composites produced were homogeneous, as evidenced by the slight deviation from the average hardness value.

**Figure 6:**
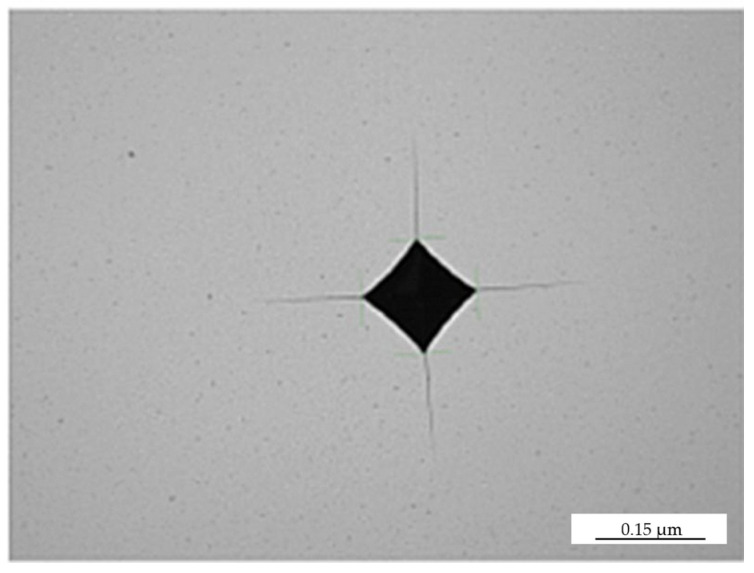
Vickers indentation on consolidated samples.

For all sample variants tested, a fracture toughness factor of 8 MPa m^1/2^ was obtained. Vickers indentations on each sample are presented in Figure 6. It was found that the fracture toughness increases with the slight increase in the WC grain size, as well as the increase in the Co mean free path, which exhibits an inverse tendency as compared with the hardness.

**Figure 7:**
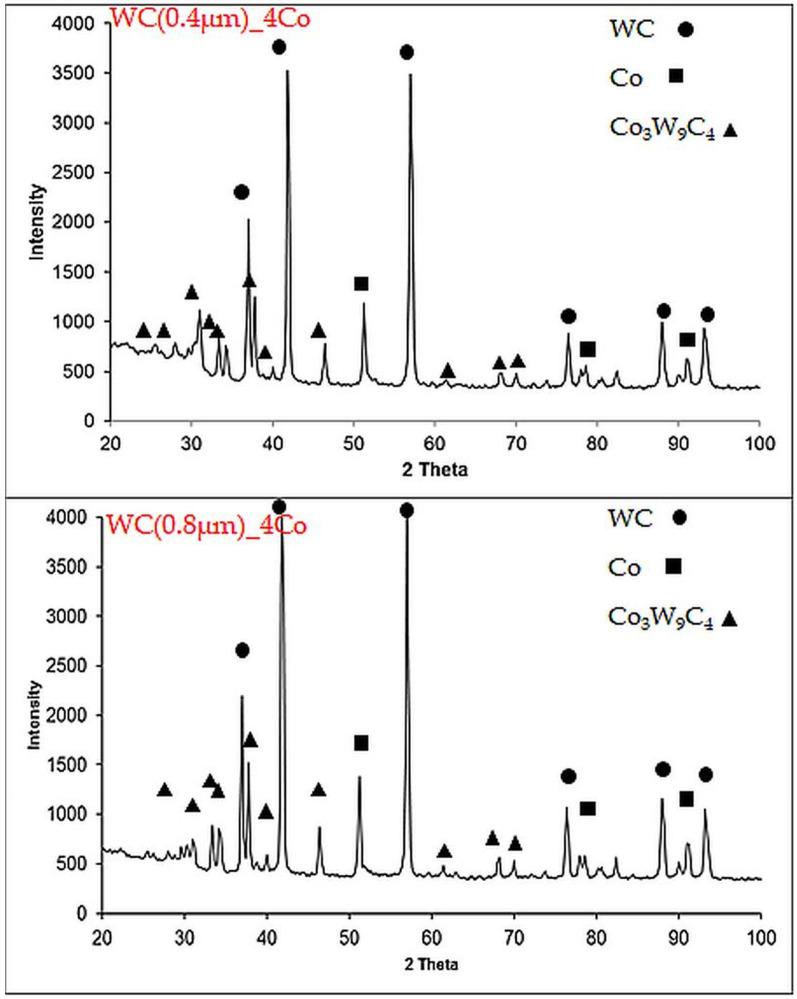
XRD patterns with a selected 2θ range of 20°–100° for WC-Co sinters (Co: 96-901-2885; WC: 96-210-2245; Co_3_W_9_C_4_: 96-152-8858).

In order to determine the phase composition of the carbides, XRD analysis was carried out for the resulting WC-Co sinters, as shown in Figure 7. It is shown that strong diffraction peaks of the WC, FCC Co and Co_3_W_9_C_4_ are observed in all sinters.

Previous investigations have indicated that FCC Co is equipped with more sliding systems than HCP Co; the dislocations in FCC Co are easier to slide when external forces are applied, and thus higher toughness can be achieved [35,36].

It can be concluded that a ternary carbide phase (η), such as Co_3_W_9_C_4_, appears in this region by mutual diffusion. This η phase is generated when C-deficient W dissolves into Co. Unfortunately, depending on the volume with the η phase, the mechanical properties of WC-Co may deteriorate. 

The authors in [37] carried out the sintering process of W-6%Co-C powder mixtures at the temperature of 1500 °C under pressure. Studies have shown that the type of phases obtained depends on the relative content of carbon and cobalt. With a carbon content of 7% by mass, the WC monocarbide forms alone, and densification occurs in the presence of a cobalt-based liquid phase. With lower carbon contents, the hemicarbide W_2_C forms first and reacts with cobalt to form the mixed carbide Co_3_W_9_C_4_. In contrast, the authors of [38] prepared a new WC-Co base hardmetal by using Co, W, C, a complex carbide such as Co3W9C4 (K) and Co2W4C (θ) as the main starting material. The obtained sinters had 10–15% higher fracture toughness than conventional alloys with identical hardness. However, the transverse-rupture strength was comparable to that of the conventional alloy.

According to previous studies [39], during the carbonization process, the Co_3_W_9_C_4_ powder phase is first converted to Co_3_W_3_C, according to the reaction:

Co_3_W_9_C_4_ + 3C → 6WC + Co_3_ W_3_C (3)

Then, Co_3_W_3_C could further react with C to form WC and Co below 1230 °C. Furthermore, the W or W_2_C phase could react with C to form WC:

Co_3_W_3_C + 2C → 3WC + 3Co (4)

W_2_ C + C → 2WC (5)

W + C → WC (6)

The measured properties of the WC-4Co composites sintered using the U-FAST method are shown in Table 4 and compared with literature studies of similar composites. 

The values of *K_IC_*, calculated based on the Palmqvist fractures, also correspond to already published studies. Fang reported *K_IC_* values ranging from 8.8 to 9.5 MPa m^1/2^ for hardness range from 1887 to 2084 HV [42].

In general, an increase in the hardness of carbides translates simultaneously into a decrease in fracture toughness. However, it was shown in [43] that for nanostructured composites, fracture toughness does not decrease with increasing hardness. Even for samples having submicron grain sizes in the sintered state, there is rather a trend towards an improvement in the hardness/ductility ratio. 

Furthermore, it can be seen from Table 2 that for the sample obtained using the SPS technique, with a grain size of 0.227 µm and a hardness of 2320 HV30, the fracture toughness is very high. However, the method of calculating the *K_IC_* should be noted here—the Charles and Evan relationship was used here. *K_IC_* values calculated according to different equations, unfortunately, are not consistent and vary significantly. Of the test results analyzed, summarized in Table 4, the sinters with the higher Co content of 9.3 MPa m^1/2^ and the smallest WC granulation showed the highest *K_IC_* value. 

Attention should also be drawn to the composites obtained using the conventional liquid phase sintering method, despite the lower gradation of the powder used—0.200 µm—a lower hardness was obtained compared to sinters consolidated using the U-Fast technology.

## 5. Conclusions

The U-FAST technology used in this study allowed us to obtain materials with a homogeneous microstructure and high hardness.

In the sintering processes, only commercial WC-Co powder was used, without additives that inhibit grain growth. Already at a temperature of 1200 °C, sinters with a density close to 100% of the theoretical density were obtained. Comparison of the obtained test results with other works showed that the WC-Co composites obtained by the U-FAST method are characterized by high hardness (2270 ± 18.9 HV30 for WC(0.4 µm)_4Co sinters and 2085 ± 22.9 for HV30 WC(0.8 µm)_4Co sinters) and relatively good fracture toughness (about 8 MPa m^1/2^). Further optimization of the process parameters will make it possible to obtain sinters with even better parameters.

## Figures and Tables

**Table 1:** Parameters of the U-FAST device [27].

Max. pulse current (kA)	10
Max. output voltage (V)	14
Pulse duration (ms)	0.8–500
Temperature measurements (°C)	0–2500
Vacuum (mbar)	10^−2^–10^−6^
Max. pressing force (kN)	350
Max. sintered components	Ø 85
Max. diameter of graphite die (mm)	200
Additional pause (ms)	0–999
Number of pulses	1–500

**Table 2:** Cobalt powder composition.

Powder	Carbon wt%	Copper wt%	Iron wt%	Nickel wt%	Oxygen wt%	Silver wt%	Sulfur wt%
Cobalt	0.15	0.0050	0.020	0.30	0.60	0.0030	0.010

**Table 3:** Properties of the WC powder examined in this study.

Property	0.4 µm WC	0.8 µm WC
Total C (wt%)	6.16	6.15
Free C (wt%)	<0.08	0.03
Al (ppm)	50	<10
Ca (ppm)	50	1
Cr (ppm)	200	18
V (ppm)	280	-
Fe (ppm)	200	86
Mo (ppm)	50	5
Si (ppm)	50	-

**Table 4:** Comparison of mechanical properties of WC-Co sintered in this study with previously reported values.

Grain Size (µm)	Cobalt Content (% wt.)	Hardness (HV30)	*K_IC_* (MPa m^1/2^)	Technology
0.4	4	2270	8.33 (Schetty)	U-FAST
0.8	4	2085	8.36 (Schetty)	U-FAST
0.227	4	2320	10.45 (Charles and Evan)	SPS [34]
0.200 µm	4	2140.1	8.62 (Schetty)	conventional liquid phase sintering in hydrogen [40]
0.5	5	1886	9.3 (Schetty)	No data [41]

## Data Availability

Not applicable.
